# 

**Published:** 1897-11

**Authors:** 


### NOTICES.

### Indiana State Dental Association.

The Indiana State Dental Association will meet in the Summer of 1898 at the time and place designated by Ohio for holding the Tri-State meeting. A business meeting of the Board af Trustees will be held on the last Tuesday in June at Indianapolis.

*President*—S. B. Hartman, Ft. Wayne.

*Vice-President*—J. M. Teal, Kendallville.

*Treasurer*—R. T. Oliver, Indianapolis.

*Secretary and Chairman Tri-State Committee*—G. E. Hunt, Indianapolis.

G. E. Hunt, *Secretary.*

### EDITORIAL.

### The Ohio State Dental Society.

Below is given a notice from the Chairman of the Executive Committee. The aim is to make this meeting one of the best that has ever been held, and from information from various sources, this effort is likely to be crowned with success.

This society has a history of which its members may well be proud, for it has done much especially in its earlier times, for the progress of dentistry in this State. The change brought about by the Union of the American Dental Association, and the Southern Association, is one that interests State societies very much. That Union contemplates a more deffinite relationship between the National society and the State organizations. This is a matter that ought to be considered by the State societies. Within a short time the full program will be issued. It is very important to have a large meeting.

"The annual meeting of the Ohio State Dental Society will be held at Neil House, Columbus, O., December 7th, 8th, and 9th.

This promises to be one of the banner meetings of this flourishing society. It has been the aim of the Executive Committee for the last few years to have new names on the program each meeting. The same scheme is being carried out this year. A new feature for this year will be an all day clinic. Many interesting things are promised.

J. R. Callahan,

*Chairman of Executive Commiteee.*"

### CONTENTS.

COMMUNICATIONS.

### THE DENTAL REGISTER,

**A MONTHLY MAGAZINE DEVOTED TO THE INTERESTS OF THE**

### DENTAL PROFESSION.

*Terms Reduced to* $2.00 *Per Year. Single Copies,* 25 *Cents.*

**EDITED BY PROF. J. TAFT, D.D.S.**

—AND—

**Published by SAM'L A. CROCKER & CO., Ohio Dental and Surgical Depot.**

The volume commences in January and closes in December.

The REGISTER being issued monthly is always fresh, therefore Indispensable to the practitioner who desires to keep posted up with the new inventions and improvements which are daily developed. Neither expense nor effort will be spared to give value and variety to its contents. Articles will be illustrated with well executed engravings whenever they will add to the interest or assist to explanation.

Its extensive circulation and frequency of issue make it one of the BEST DENTAL ADVERTISING MEDIUMS in the United States.

All subscriptions must begin with January or July.

Local or special notices, five lines or less, will be inserted for $3 each insertion ; over five lines, 50 cts. per line.

All advertising bills payable in advance, or at furthest, after the issue of the first number containing the advertisement. All transient advertisements to be paid for in advance.

☞CONTRIBUTIONS SOLICITED.

Exclusively Editorial Communications should be addressed to the Editor.

The latest number of the REGISTER may be ordered with or without other good [illegible] any time, and all letters should be addres[illegible]

